# Vitamin D Levels Are Inversely Associated with Liver Fat Content and Risk of Non-Alcoholic Fatty Liver Disease in a Chinese Middle-Aged and Elderly Population: The Shanghai Changfeng Study

**DOI:** 10.1371/journal.pone.0157515

**Published:** 2016-06-10

**Authors:** Dan Wang, Huandong Lin, Mingfeng Xia, Qiqige Aleteng, Xiaoming Li, Hui Ma, Baishen Pan, Jian Gao, Xin Gao

**Affiliations:** 1 Department of Endocrinology and Metabolism, Shanghai Zhongshan Hospital, Institute of Chronic Metabolic Diseases, Fudan University, Shanghai 200032, China; 2 Department of Laboratory Medicine, Zhongshan Hospital, Fudan University, Shanghai, 200032 China; 3 Department of Clinical Nutrition, Zhongshan Hospital, Fudan University, Shanghai, 200032 China; Indiana University Richard M. Fairbanks School of Public Health, UNITED STATES

## Abstract

**Background/Objectives:**

Vitamin D exerts metabolic activities. We investigated whether the 25-hydroxy vitamin D [25(OH)D] is associated with liver fat content (LFC) and non-alcoholic fatty liver disease (NAFLD) in a middle-aged, elderly Chinese population.

**Subject/Methods:**

A total of 2,960 participants (954 men and 2,006 women) aged over 45 years old were enrolled. Each participant underwent a standard interview, anthropometric measurements and laboratory examinations. Vitamin D deficiency and insufficiency was diagnosed when serum 25(OH) D level was < 50 and 50–75nmol/L. An ultrasound quantitative method was used to assess the LFC.

**Results:**

Among the 2,960 participants, 1,982 (67.0%) subjects had vitamin D deficiency, 769 (26.0%) had vitamin D insufficiency, and 209 (7%) had normal vitamin D. Male subjects with vitamin D deficiency and insufficiency had significantly higher LFC than those with normal 25(OH)D (P = 0.034), while the LFC values showed no significant difference among the female subjects with vitamin D sufficiency, insufficiency and deficiency (P = 0.396). Univariate correlation analysis showed that 25(OH)D had a significantly negative association with LFC in men (r = -0.085, P = 0.009), but not in women. After adjusting for age, cigarette smoking, examination season, serum calcium, PTH and all possible confounders that displayed significant associations with LFC in univariate correlation analysis, serum 25(OH)D remained associated with LFC in middle-aged and elderly Chinese men.

**Conclusion:**

Serum 25(OH)D level was inversely associated with LFC in middle-aged and elderly Chinese men.

## Introduction

Non-alcoholic fatty liver disease (NAFLD) is a pathological condition that consists of a spectrum of liver diseases caused by macrovesicular accumulation of triglycerides within hepatocytes (hepatic steatosis). Currently, NAFLD is thought to be the hepatic manifestation of insulin resistance and correlated with metabolic syndrome, diabetes and cardiovascular diseases [1]. The prevalence of NAFLD is approximately 10–30% in the general population, and it is still increasing worldwide [2,3]. Detection of novel risk factors could promote our understanding of the NAFLD pathogenesis, and facilitate early diagnosis and treatment of this disease clinically.

Vitamin D plays an important role in calcium and phosphorus metabolism, and recent studies have focused on the extra-skeletal effects of vitamin D. Vitamin D has been implicated in many metabolic disorders, such as type 2 diabetes, insulin resistance, metabolic syndrome and cardiovascular diseases [4–7], and experimental studies showed that Vitamin D can improve free fatty acid (FFA)-induced insulin resistance in peripheral tissues and hepatocytes [8].

Several recent studies had shown a relationship between presence of NAFLD and serum 25-hydroxy vitamin D [25(OH)D] level [9]. However, most of the previous studies used common ultrasound method to diagnose NAFLD, which provided no information on the severity of liver steatosis. The quantitative association between liver fat content (LFC) and serum 25(OH)D has not been fully studied.

In the current study, we measured LFC by a ultrasound quantitative method in a large-scale middle-aged and elderly Chinese population, and investigated whether liver fat accumulation was associated with serum 25(OH)D level.

## Subjects and Methods

### Subjects

The Changfeng Study is a community-based study of chronic diseases among middle-aged and elderly individuals, which has been described previously [10].A total of 4659participants aged over 45 years oldwereinitially recruitedfrom October 2010 to July 2012. Exclusion criteria were incomplete data of serum 25(OH)D or LFC (n = 472), alcohol abuse (>20 g/day for male, >10 g/day for female) [11] (n = 581), chronic viral hepatitis (HbsAg or anti-HCVAb positive) (n = 261), history of viral, autoimmune or drug-induced chronic hepatitis, cirrhosis, or liver cancer (n = 385) Finally, 2960 subjects (954 males and 2006 females) were included in the analysis.

The study was approved by the ethics committee of Zhongshan Hospital at Fudan University, and it was done in accordance with the guidelines of Declaration of Helsinki. Each subject provided written informed consent. Interviews, physical examinations and ultrasound scans were performed at the Changfeng Community Health Service Center.

### Ultrasound quantitative examinations

Trained ultrasonographists who were unaware of clinical data performed ultrasound examinations. Ultrasound images were captured using a GE logiq P5 scanner (GE Healthcare, Milwaukee, WI, USA), analyzed using a NIH image software (ImageJ 1.41o, NationalInstitutes of Health, Bethesda, MD) and standardized using a tissue-mimicking phantom (Model 057; Computerized Imaging Reference Systems, Norfolk, VA). LFC was calculated using the equation: LFC (%) = 62.592 * standardized US hepatic/renal ratio + 168.076*standardized US hepatic attenuation rate—27.863 [12,13].

### Other Measurements

Participants were required not to alter their diets or physical activity levels for at least 3 days before the test. Trained investigators interviewed all participants to evaluate the medical history and lifestyle. The time of examination was divided into four subgroups: (1) spring (March-May), (2) summer (June-August), (3) autumn (September-November), and (4) winter (December-February). Weight and height were measured while each participant was clothed in a light gown. The body mass index (BMI) was calculated asweight divided by height squared (kg/m^2^). Waist circumference (WC) was measured midway between the lowest rib margin and the iliac crest in a standing position. The resting blood pressure was measured three times using a standardized sphygmomanometer after five minutes of rest, and the mean value was used for the analysis. All of the subjects were examined after an over-night fast. Total cholesterol (TC), high-density lipoprotein cholesterol (HDL-C), triglycerides (TG), liver enzymes, and serum calcium and phosphate were measured using a model 7600 automated bio-analyser (Hitachi, Tokyo, Japan). The level of low-density lipoprotein cholesterol (LDL-C) was calculated using the Friedewald equation. The fasting blood glucose (FBG) and 2h postload blood glucose (PBG) following a 75-g oral glucose challenge were measured using the glucose oxidase method. Hemoglobin A1c (HbA1c) was measured by high performance liquid chromatography (HPLC) (BIO-RAD II TURBO), which was standardized to the National Glycated Haemoglobin Standardization Program (NGSP). The serum 25(OH)D, parathyroid hormone (PTH) and fasting insulin (FINS) were measured by electrochemiluminescence immunoassay using an immunoassay analyzer (Roche Cobas-6001, Switzerland). The homeostasis model assessment insulin resistance index (HOMA-IR) was obtained by applying the following formula: HOMA-IR = fasting insulin (mU/L) × fasting glucose (mmol/L)/22.5[14].

### Diagnosis of metabolic syndrome

Metabolic syndrome was defined as follows: waist circumference≥90 cm for males, and _≥80 cm for females, antihypertensive treatment or blood pressure≥130/85 mmHg, antidiabetic treatment or FBG≥5.6 mmol/L, TG ≥1.7 mmol/L, and HDL cholesterol <1.03 mmol/L for males and <1.29 mmol/L for females [15].

### Statistical analyses

All statistical analyses were performed using SPSS software version15.0 (SPSS, Chicago, IL).The data were presented as the mean±SD, except for skewed variables, which were presented asthe median with the interquartile range (25–75%) given in parentheses. One-way analysis of variance or the Mann–Whitney U-test was used for the comparisons of continuous data among groups, whereas the Chi-squared test was used for the comparisons of categorical variables. The correlations of LFC with all anthropometric and biochemical parameters were analyzed by Spearman correlation analysis. All subjects in the community were divided into three subgroups according the level of serum 25(OH)D: Vitamin D sufficiency (> 75nmol/L), insufficiency (50–75nmol/L)and deficiency(<50nmol/L) [16]. Independent associations between the 25(OH)D categories and LFC were assessed using five multivariate linear regression models, after adjusting for age, cigarette smoking, examination season, serum calcium, PTH and all variables that displayed significant associations with LFC in univariate correlation analysis. A two-tailed P-value < 0.05 was considered statistically significant.

## Results

### General characteristics of the study population

A total of2960 participants (954 men and 2006 women), 46–96 years of age, were enrolled for the analyses. Their baseline characteristics are given in Table 1. The mean BMI was 24.2±3.3kg/m^2^ and median LFC was 5.3% (2.4%-11.6%). Among the 2960 participants, 1982 (67.0%) subjects had vitamin D deficiency, 769 (26.0%) had vitamin D insufficiency, and 209 (7%) had normal vitamin D. Compared with subjects with sufficient vitamin D, subjects with decreased serum 25(OH)D were younger, more obese, and had significantly higher blood glucose, fasting insulin, cholesterol, LDL-c, bone turn-over biomarkers (PTH and ALP), and LFC (All p value <0.05). The prevalence of NAFLD in subjects with vitamin D sufficiency, insufficiency and deficiency was 24.4%, 31.2% and 33.8%, respectively (P = 0.016).

**Table 1:** Characteristics of the study participants according to vitamin D levels.

	Vitamin D			
	Sufficiency (>75nmol/L)	Insufficiency (50–75nmol/L)	Deficiency (<50nmol/L)	P value
N	209	769	1982	
Gender (M/F)	120/89	304/465	530/1452	<0.001
Smoking, n(%)	29 (13.9%)	113 (14.7%)	239 (12.1%)	0.223
Examination season				<0.001
Spring, n (%)	791 (39.9%)	223 (29.0%)	37 (17.7%)	-
Summer, n (%)	405 (20.4%)	216 (28.1%)	73 (34.9%)	-
Autumn, n(%)	398 (20.1%)	225 (29.3%)	67 (32.1%)	-
Winter, n(%)	388 (19.6%)	105 (13.7%)	32 (15.3%)	-
Age	65.27±9.86	64.57±9.65	62.82±9.73**	<0.001
BMI	23.46±2.86	24.19±3.22**	24.25±3.41**	0.005
WC	81.91±8.76	83.44±9.36*	82.99±9.73	0.118
SBP	134.71±20.20	135.14±18.02	136.29±19.97	0.256
DBP	75.36±10.15	75.18±9.50	76.52±9.90	0.003
FBG	5.0(4.8–5.5)	5.2(4.9–5.8)**	5.2(4.8–5.8)**	0.001
PBG	6.6(5.5–7.8)	6.9(5.8–8.7)**	6.7(5.5–8.6)*	0.011
HbA1c	5.6(5.4–5.9)	5.7(5.4–6.1)*	5.7(5.4–6.0)**	0.022
FINS	6.7(4.9–9.7)	7.9(5.5–11.3)	8.2(5.5–11.9)*	0.040
HOMA-IR	1.79±1.99	2.45±2.78	2.75±4.98*	0.024
TG	1.29(0.96–1.69)	1.46(1.1–2.0)	1.44(1.06–2.05)	0.173
TC	4.88±0.97	5.08±0.89 **	5.16±0.94**	<0.001
HDL-c	1.43±0.39	1.42±0.36	1.44±0.37	0.185
LDL-c	2.78±0.75	2.90±0.77	2.94±0.81**	0.017
Cr	73(61–87)	67(58–80) **	63(55–73)**	<0.001
Ca	2.42(2.34–2.50)	2.4(2.3–2.5)	2.38(2.3–2.46)	0.558
P	1.10(0.98–1.20)	1.1(1.0–1.2)	1.15(1.04–1.24)	0.612
PTH	32.3(26.3–41.5)	37.3(29.5–47.9) **	43.7(33.8–55.0)**	<0.001
ALT	16(13–20)	16(12–22)	15(12–21)	0.627
AST	20(18–24)	20(17–24)	20(17–23)	0.758
ALP	68(59–79)	71(61–83.5)	72(61–86)**	0.009
GGT	22(17–31)	22(17–32)	22(17–32)	0.508
LFC	3.83(1.90–8.99)	5.06(2.10–11.42)	5.59(2.58–11.76)**	0.002
NAFLD, %	24.4	31.2	33.8	0.016

Data are expressed as the means ± SD, percentages or median (25th to 75th percentiles).

*P < 0.05

**P < 0.01 vs. Vitamin D sufficiency group.

### Association between serum 25(OH)D and NAFLD

**Fig 1:**
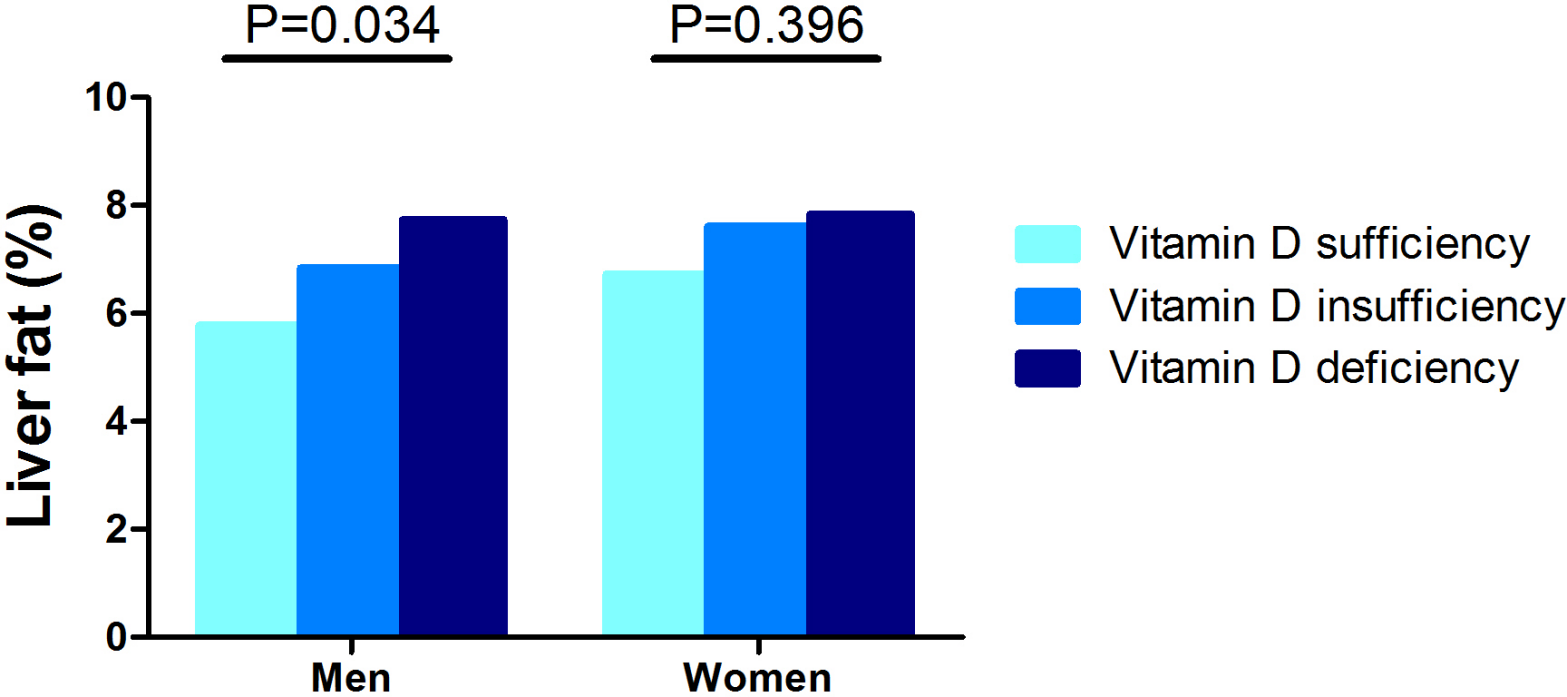
The average LFC (%) in male (left) and female (right) subjects with different vitamin D status with a serum 25(OH)D <50nmol/L, = 50–75nmol/L and >75nmol/L. The liver fat content was significantly higher in male subjects with vitamin D deficiency and insufficiency, but not in female subjects.

By taking the gender difference of vitamin D status into consideration [17], we stratified all analyses by sex. As shown in Fig 1, male subjects with vitamin D deficiency and insufficiency had significantly higher LFC than those with normal 25(OH)D (P = 0.034), while the LFC values showed no significant difference among the female subjects with vitamin D sufficiency, insufficiency and deficiency (P = 0.396). Linear correlation analyses also showed that 25(OH)D had a significantly negative association with LFC in men (r = -0.085, P = 0.009), but not in women (r = -0.014, P = 0.523) in our middle-aged and elderly study population (Table 2).

**Table 2:** Correlations between LFC, 25(OH)D and other metabolic parameters.

	Men		Women	
	r	P	r	P
age	-0.095	0.003	-0.026	0.243
Smoking	0.045	0.164	-0.002	0.936
Season	0.006	0.855	0.001	0.952
BMI	0.364	<0.001	0.335	<0.001
WC	0.349	<0.001	0.297	<0.001
SBP	0.038	0.024	0.118	<0.001
DBP	0.111	0.001	0.156	<0.001
TC	0.117	<0.001	0.062	0.005
TG	0.194	<0.001	0.288	<0.001
HDL	-0.185	<0.001	-0.225	<0.001
LDL	0.089	0.006	0.000	0.990
FBG	0.105	0.001	0.186	<0.001
PBG	0.201	<0.001	0.262	<0.001
HbAlc	0.096	0.005	0.211	<0.001
FINS	0.067	0.038	0.298	<0.001
HOMA-IR	0.053	0.101	0.265	<0.001
Cr	-0.078	0.016	-0.070	0.002
Ca	-0.010	0.758	0.007	0.755
P	0.046	0.152	-0.011	0.614
25(OH)D	-0.085	0.009	-0.014	0.523
PTH	-0.101	0.002	0.007	0.762
Menopause	-	-	0.034	0.126

### Univariate analyses on the associations between metabolic parameters and NAFLD

As shown in Table 2, LFC was significantly associated with BMI, all components of metabolic syndrome (waist circumference, blood pressure, serum TG, HDL-c, FBG, and PBG), serum insulin and HbA1c in both male and female subjects (all p <0.05). In parallel to the change of 25(OH)D, serum PTH (r = -0.101, P = 0.002) and creatine levels (r = -0.078, P = 0.016) were also negatively associated with LFC in men, but in women, LFC had no significantly associations with bone metabolic parameters, including serum calcium, phosphate, PTH, and 25(OH)D.

### Independent contribution of 25(OH)D in variability in LFC

In unadjusted linear regression analysis, 25(OH) was significantly correlated with LFC (stdβ = -0.084, P = 0.010). In multiple regression analyses, after adjustment for age and cigarette smoking (model 1), 25(OH)D was still significantly associated with LFC (stdβ = -0.074, P = 0.024). Parameter estimates of these correlations remained significant after successively adjusting for examination season (model 2), serum calcium, PTH and creatine (model 3), and then in addition BMI (model 4). In model 5, the significant negative correlation between LFC and 25(OH)D remained after additional adjustment for all components of metabolic syndrome, including WC, FBG, SBP, TG and HDL-c, and insulin resistance level measured by HOMA-IR (stdβ = -0.074, P = 0.016) (Table 3).

**Table 3:** Multivariate regression analysis for the association between liver fat content (dependent variable) and 25(OH)D categories (independent variables) in different models in men.

	Liver fat content			
	Std β	β	95%CI	P
Unadjusted	-0.084	-0.954	(-1.675, -0.233)	0.010
Model 1	-0.074	-0.836	(-1.562, -0.111)	0.024
Model 2	-0.076	-0.859	(-1.595, -0.123)	0.022
Model 3	-0.092	-1.051	(-1.789, -0.312)	0.005
Model 4	-0.083	-0.938	(-1.626, -0.249)	0.008
Model 5	-0.074	-0.859	(-1.558, -0.160)	0.016

Model 1: adjusted for age and cigarette smoking

Model 2: Model 1 plus season

Model3: Model 2 plus serum creatine, calcium and PTH

Model4: Model 3 plus BMI

Model 5: Model 4 plus all components of metabolic syndrome (waist circumference, FBG, SBP, TG and HDL-c) and HOMA-IR.

## Discussion

In this study, we found that 1) individual vitamin D deficiency or insufficiency was associated with impaired glucose and lipid metabolism and higher prevalence of metabolic syndrome; 2) serum 25(OH)D concentration had an independent and negative association with LFC and the presence of NAFLD in middle-aged and elderly Chinese men, but not in women. To the best of our knowledge, this is the first study to investigate the relationship between serum 25(OH)D and LFC which is measured by quantitative ultrasonography.

At present, ultrasonography remains the most common method for the diagnosis of liver steatosis, because it is simple, non-invasive, inexpensive, and widely available [18]. However, the conventional ultrasonography is limited due to its poor sensitivity and inability to quantify LFC [19]. While the current accurate quantitative modalities, such as liver biopsy or proton magnetic resonance spectroscopy ([^1^H]-MRS), can not be carried out routinely in the large-scale cross-sectional studies due to either the invasiveness [20] or the demanding technologies and expensive costs [18, 21]. Therefore, there are still few epidemiological evidences on the quantitative correlation between LFC and serum 25(OH)D level until now. Recently, several new techniques have been developed to facilitate the measurement of LFC in large-scale community population, including the controlled attenuation parameter (CAP) [22] and backscatter coefficient [23], but these methods depend on the specialized equipment that was not routinely used in clinic. In the current community study, we used a method of computer-aided ultrasound measurement of LFC based on the ultrasound hepatic/renal ratio and hepatic attenuation rate under traditional ultrasonic apparatus [12]. The accuracy of the quantitative ultrasound examination has been validated using [^1^H]-MRS as a reference [12] and was consistent with both [^1^H]-MRS(r = 0.89, P<0.001) and histology-determined steatosis grade (r = 0.79, P<0.001) [13]. It has also been used in our previous community studies [24–26]. Therefore, we can provide reliable quantitative relation between individual liver fat content and vitamin D status.

Recent laboratory studies have shown a complex network of interactions among adipose, liver and bone tissues [27]. Vitamin D is one of the primary biological regulators of calcium and phosphates homeostasis in the body [28]. With the wide tissue distribution of the vitamin D receptor (VDR), vitamin D can function the same way as other steroid hormones in the whole body [29]. Previous studies have revealed the role of 25(OH)D in regulating the glucose and lipid metabolism [30–32], and the cardiovascular protective action of vitamin D [33]. In accordance with the previous studies, we also found thatlow 25(OH)D was associated with impaired glucose and lipid metabolism such as higher BMI, blood pressure, blood glucose, HbA1c, FINS, HOMA-IR, TC and LDL-C levels.

NAFLD is regarded as the liver manifestation of the metabolic syndrome [1].Several studies have reported the association between low serum 25(OH)D concentrations and NAFLD in humans. Targher et al. have compared 60 consecutive patients with biopsy-proven NAFLD and 60 healthy controls of comparable age, sex and BMI, and the NAFLD patients had a remarkable decrease in serum 25(OH)D concentrations [34]. Zhai HL et al. showed a negative correlation between vitamin D level and NAFLD diagnosed by common ultrasonography in a large-scale community study [35]. A meta-analysis of 17 cross-sectional and case-control studies concluded that NAFLD patients were 1.26 times more likely to be vitamin D deficient [36]. Recently, Nelson JE et al. also reported a relationship between vitamin D deficiency and increased risk of non-alcoholic steatohepatitis diagnosed by liver biopsy [37]. Our study further expand the knowledge on the association between vitamin D status and NAFLD, and importantly evaluated the independently inverse relationship between the quantitative value of LFC and serum 25(OH)D level.

The mechanisms underlying the inverse relation between NAFLD and 25(OH)D are still not clear. On the one hand, vitamin D was converted to 25(OH)D in the liver for its active form [38], However, most of NAFLD patients in our study had normal liver enzyme, albumin level and coagulation function, which indicated a normal liver synthesis function of 25(OH)D. On the other hand, vitamin D also had extra-skeletal effects on metabolic organs, which may indirectly affect the hepatic lipid metabolism [28]. The role of vitamin D in adipokine activity is a current active area of research, Roth et al. has investigated the effect of vitamin D deficiency on adiponectin, leptin, resistin, TNF-α, and IL-6 [39], and it may worsen NAFLD by up-regulation of hepatic inflammatory and oxidative stress genes. In addition, it is reported that vitamin D receptor was widely expressed in the liver, and the negative association between the severity of liver histology and vitamin D receptor expression in NASH patients indicated that vitamin D might have a direct role in the progression of NAFLD [40].

Interestingly, we found a relationship between LFC and 25(OH)D in middle-aged and elderly men, but not in women. The gender difference has also been found in our previous study on the association between LFC and bone mineral density in the same community population [41]. The difference between men and women in the features of age-related bone metabolism, liver lipid metabolism and sex hormone levels could be potential explanations. However, we cannot deduce the mechanisms of the gender difference in the association between LFC and 25(OH)D in our current study, which need to be elucidated further.

Our study has some limitations. Firstly, the present study was across-sectional study, and did not permit the identification of causal relationships between LFC and 25(OH)D, which need to be further evaluated in longitudinal studies. Secondly, in a study of our current size, it was not possible to obtain liver biopsies to investigate the association of 25(OH)D with liver fibrosis or inflammation. Thirdly, the estrogen and androgen levels have not been measured in the current study, which might be responsible for the gender difference in the relationship between 25(OH)D and NAFLD.

## Conclusion

Serum 25(OH)D was inversely associated with LFC in middle-aged and elderly Chinese men. Vitamin D replacement may have benefits against NAFLD and liver fat accumulation, which need to be validated by further well-designed clinical trials in the future.
